# Assumptions about the positioning of virtual stimuli affect gaze direction estimates during Augmented Reality based interactions

**DOI:** 10.1038/s41598-019-39311-1

**Published:** 2019-02-22

**Authors:** Nicola Binetti, Tianchang Cheng, Isabelle Mareschal, Duncan Brumby, Simon Julier, Nadia Bianchi-Berthouze

**Affiliations:** 1 UCL Interaction Centre, University College London, London, UK; 2 School of Biological and Chemical Sciences, Psychology, Queen Mary University of London, London, UK; 3 Department of Computer Science, University College London, London, UK

## Abstract

We investigated gaze direction determination in dyadic interactions mediated by an Augmented Reality (AR) head-mounted-display. With AR, virtual content is overlaid on top of the real-world scene, offering unique data visualization and interaction opportunities. A drawback of AR however is related to uncertainty regarding the AR user’s focus of attention in social-collaborative settings: an AR user looking in our direction might either be paying attention to us or to augmentations positioned somewhere in between. In two psychophysical experiments, we assessed what impact assumptions concerning the positioning of virtual content attended by an AR user have on other people’s sensitivity to their gaze direction. In the first experiment we found that gaze discrimination was better when the participant was aware that the AR user was focusing on stimuli positioned on their depth plane as opposed to being positioned halfway between the AR user and the participant. In the second experiment, we found that this modulatory effect was explained by participants’ assumptions concerning which plane the AR user was focusing on, irrespective of these being correct. We discuss the significance of AR reduced gaze determination in social-collaborative settings as well as theoretical implications regarding the impact of this technology on social behaviour.

## Introduction

Gaze behaviours carry important nonverbal information that inform and regulate interactions between individuals^1–3^. Mutual gaze (when we make eye contact with another person) is a precursor to most social exchanges, while averted gaze (when gaze is directed away from the other person) can signal the presence of environmental stimuli of potential interest, providing a behavioural channel for joint attention^4–6^. The biological relevance of gaze is reflected in people’s extraordinary ability of evaluating eye and head orientation and identifying eye contact^7,8^, which are enabled by dedicated neural machinery. Human imaging research^9–11^ reveals functional specialization to head and eye directional inputs in the posterior Superior Temporal Sulcus (STS) and the Inferior Temporal Lobule. More specifically, studies highlight that the STS pools eye and head directional signals to inform estimates of the other’s direction of attention^12,13^.

People’s proficiency at evaluating other’s focus of attention can be assisted by the use of various forms of technology. For example, a laser pointer can aid a public speaker’s presentation by highlighting his/her focus of attention on projected slides. On the other hand, other technologies can undermine this ability. Video conferences can introduce aspects of ambiguity regarding the other’s focus of attention, given that web cameras are positioned above the screen and that participants do not share the same physical space. An example of this is also provided by wearable augmented Reality (AR) technologies. AR systems are increasingly becoming relevant in everyday life, and while their use can provide substantial benefits across a variety of individual or collaborative activities, they can potentially introduce elements of visual uncertainty in an external viewer. If we see a person wearing an AR visor, we might wonder whether they are looking at real world stimuli or computer-generated graphics, and if that person is looking in our direction, we might wonder whether they are paying attention to us or to an augmentation positioned somewhere in between. Given these elements of visual uncertainty, to what extent do our expectations concerning an AR user’s focus of attention affect our ability of accurately interpreting their gaze behaviours? Here we studied the interaction between gaze direction estimates and an observer’s assumptions of the positioning of virtual stimuli attended by an AR user.

**Figure 1:**
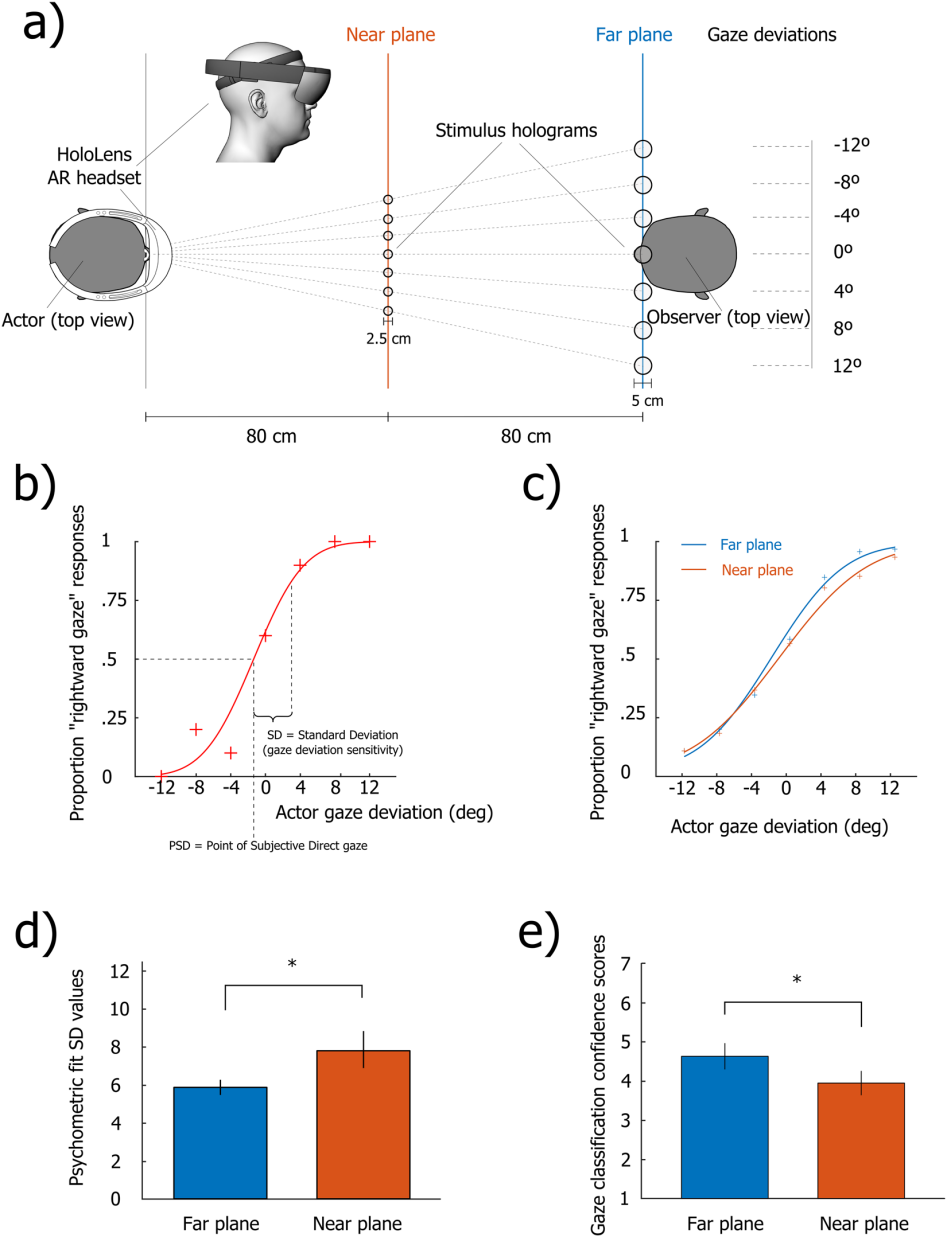
(**a**) Experimental setup. On each trial the Actor (HoloLens user) was asked to fixate on 1 of 14 possible holographic spherical stimuli, displayed at two depths (Near and Far planes) and seven levels of horizontal deviation. The Observer classified the Actor’s gaze as being leftward or rightward, relative to a direct fixation. Participants swapped Actor/Observer roles across blocks. (**b**) Psychometric fit of participant “rightward gaze” responses as a function of the Actor’s degrees of gaze deviation. We extracted the 50% point (PSD = Point of Subjective Direct gaze; measure of bias in perceived gaze direction) and the standard deviation (SD = standard deviation; measure of gaze direction sensitivity) of the underlying Gaussian distribution. (**c**) Pooled data psychometric fits for gaze direction classifications on Near and Far planes. (**d**) Average SD values for Near and Far planes. (**e**) Average confidence scores (1 = not confident at all −7 = fully confident) for performance in gaze classification task. Error bars depict the Standard Error of the Mean (SEM).

We addressed this question in two psychophysical experiments by studying gaze interactions between participant pairs, mediated by a Microsoft HoloLens AR headset (https://www.microsoft.com/en-gb/HoloLens). Each pair involved one participant (the ‘Actor’) wearing the HoloLens routinely fixating on a set of holograms, and another participant (the ‘Observer’) performing gaze direction classifications of the Actor’s fixation behaviours. We measured whether the Observer’s gaze discrimination performance was affected by his/her awareness or assumptions regarding the positioning of holographic stimuli attended by the Actor (i.e. whether the Actor fixated on stimuli positioned halfway between the pair or on the same plane occupied by the Observer). We had the Actor fixate on a set of holographic stimuli, horizontally arranged at various degrees of deviation relative to the Observer’s midline and positioned at 2 depths: halfway between participants (termed Near plane) or on the same plane occupied by the Observer (termed Far plane) (Fig. 1a). On each trial the Actor fixated on one stimulus displayed at a given deviation and on a given depth plane, while the Observer indicated with a binary response whether the Actor’s gaze was pointing leftwards or rightwards, relative to a direct fixation. We also factored in participant gender which has been previously observed to modulate gaze behaviour directed towards the eye region^14^, and could in theory modulate gaze discrimination performance.

In a first experiment we measured the Observer’s gaze direction judgments in response to Actor’s fixations to stimuli displayed on the Far or Near planes. We informed the Observer on which plane the stimuli would be displayed ahead of time (prior to each block). We measured gaze direction sensitivity and observed improved discrimination performance when participants were aware that the AR user was attending stimuli on the Far plane. We carried out a second experiment, in which stimuli were randomly assigned to the Near or Far planes, to determine whether this effect was driven by expectations regarding the positioning of stimuli attended by the Actor, or by subtle differences in the Actor’s left/right eye vergence behaviours directed towards stimuli displayed at different depths which might aid gaze direction classifications. We tested differences in gaze discrimination sensitivity based on two different data pooling criteria: a comparison based on the plane the stimuli were factually displayed on within each trial (Objective Plane Comparison: Objective Far Vs Objective Near), or a comparison based on the plane the Observer thought the stimuli were displayed on within each trial (Subjective Plane Comparison: Subjective Far Vs Subjective Near). We observed that discrimination performance improved only when participants believed that stimuli were displayed on the Far plane, irrespective of this assumption being correct, thus demonstrating that a subjective expectation regarding the positioning of virtual content attended by the Actor (i.e. whether the Actor fixated on stimuli positioned halfway between the pair or on the same plane occupied by the Observer) modulated gaze direction sensitivity. These findings have theoretical implications in our understanding of the impact of technology on social behaviour, showing how sources of sensory uncertainty that accompany the use of AR-HMDs can impact gaze interactions. Furthermore, these findings can also provide insights for the design of AR interfaces that reduce these sources of visual uncertainty.

## Methods

### Participants

#### Experiment 1

We recruited 20 participants; 8 female & 12 male, mean age = 25.8, range 18–47 years old. Sample sizes were based on comparable number of participants tested in previous gaze direction discrimination studies^15,16^. The testing session lasted approximately 1 hour per couple (30 minutes per subject performing gaze classifications in Observer role. Actor/Observer roles were swapped across testing blocks). All participants had normal or corrected to normal vision. No participant suffered from strabismus. Informed consent was obtained from all participants prior to starting the experiment. Participants were paid £7.5 (GBP) in cash or with an e-voucher for a popular online store for their participation.

#### Experiment 2

We recruited 18 participants; 9 Female & 9 Male, mean age = 29.5, range 20–55 years old. All participants had normal or corrected to normal vision. No participant suffered from strabismus. Informed consent was obtained from all participants prior to starting the experiment. Participants were paid £7.5 (GBP) in cash for their participation.

### Apparatus

#### Experiment 1

The Experiment was conducted in a controlled testing environment, with artificial overhead lighting. Each testing session involved a participant pair, who sat 160 cm apart and facing one another. The ‘Actor’ viewed virtual white spherical stimuli through a HoloLens Augmented Reality head-mounted-display (HMD), providing directional gaze stimuli that the other participant, the ‘Observer’, had to classify by pressing a button press. Participants were randomly assigned to each pair, and did not know each other personally prior to the study.

Stimuli were aligned with the bridge of the Observer’s nose (nasion) through a Vuforia (https://www.vuforia.com/) visual marker tracking technique.

Stimulus presentation, data logging and experiment logic were implemented in Unity (https://unity3d.com/) running on the Microsoft HoloLens HMD. Responses were produced on a Bluetooth wireless keyboard (www.anker.com) linked to the HoloLens. Given that stimuli were prospectively aligned, we did not restrain the Actor’s head position in Experiment 1, as we didn’t expect differences in head movements across stimuli projected on the Near and Far planes.

#### Experiment 2

We adopted an equivalent setup in Experiment 2, with the exception of some minor differences. Stimuli were aligned through a streamlined manual positioning technique in which the HoloLens wearer was asked to carefully align a target stimulus to the bridge of the Observer’s nose by tilting their head, and confirming the positioning with a button press. While we did not restrain the Actor’s head position in Experiment 1 (as we didn’t expect differences in head movements as a function of depth plane), we used a chinrest to restrain the Actor’s head movements in Experiment 2 to categorically exclude any potential confound introduced by head movements across depth conditions.

### Task design

#### Experiment 1

On each trial, the ‘Actor’ was asked to look at a virtual white spherical stimulus (subtending approximately 1.7 degrees of visual angle) through the HoloLens HMD visor that could appear at one of 14 possible locations (on two rows with 7 stimuli each). Stimuli were horizontally arranged at 7 degrees of deviation relative to the Observer’s midline (one 0 degree central stimulus, three to the left and to the right of the centre at 4, 8 and 12 degrees, respectively). The central stimulus was aligned with the Observer’s nasion, thus positioned on the participants’ eye line. Stimuli were displayed at 2 depths: halfway between participants (80 cm: Near plane) or on the same plane occupied by the Observer (160 cm: Far plane) (Fig. 1a). Stimuli on the Near and Far planes were prospectively aligned and proportionally scaled in size, ensuring that each stimulus encompassed the same degrees of visual angle and required comparable gaze deviations across planes.

Prior to running the experiment, both participants wore the HoloLens to familiarize themselves with the device and to experience the virtual stimuli. While seated facing each other, we first showed them the 7 stimuli simultaneously displayed on the Near plane followed by the 7 stimuli on the Far plane. This was done so participants got a sense of the spatial extent covered by the stimuli, and could notice that all stimuli fell within the field of view of the Hololens. After this preliminary phase, we assigned one participant to the Actor role, and one participant to the Observer role.

Trials were blocked according to the plane on which stimuli were displayed. In the ‘Near block’ all stimuli were presented on the Near plane while in the ‘Far block’ all stimuli were displayed on the Far plane (Far block). Participants were verbally informed ahead of time which plane (Near/Far) the stimuli would appear on in the upcoming block (“In the following block the Actor will only view stimuli displayed on the Near/Far plane”). Near and Far block order was counterbalanced across participant pairs. Each experimental trial began with the presentation of a visual stimulus accompanied by a brief auditory beep emitted by the HoloLens that could be heard by both participants. The Actor was instructed to hold his/her fixation on the stimulus until it disappeared. The Observer was required to indicate on each trial using a button press whether the Actor’s eyes were pointing towards the Observer’s left (“Leftward” response, pressing the Left arrow key) or towards the Observer’s right (“Rightward” pressing the Right arrow key), relative to a direct fixation. The Actor was unaware of the Observer’s response. The stimulus disappeared and the next trial began after a 1 second interval triggered by the Observer’s response, or following a 4 second interval in the absence of the Observer’s response. After completing both blocks, participants swapped Actor and Observer roles, and performed the blocks in the same order. Participants performed 10 repetitions per stimulus deviation (70 trials per block, 140 total trials). At the end the experiment we collected subjective reports on confidence level of gaze direction estimates (how confident participants felt of their performance across the Near and Far blocks, on a 7 point Likert scale). Since in this Experiment the Actor’s head was not restrained by a chinrest, we also collected head position and rotation data sampled at 10 Hz from the HoloLens.

#### Experiment 2

Participants carried out an equivalent task with the only exception that stimuli were randomly presented on either the Near or Far planes across trials within each block, as opposed to being presented in separate blocks. On each trial we collected two responses from the Observer: 1) whether the Actor was fixating Rightward or Leftward, relative to a direct fixation (Right/Left arrow key), and 2) whether the actor was fixating on a hologram positioned on the Near or Far plane (Up/Down arrow key). The stimulus remained visible until the participant produced the second response. No time limit was used in this experiment. Participants performed 10 repetitions per stimulus deviation and plane (140 total trials). We did not log head position and rotation data since the Actor’s head movements were restrained by a chinrest.

### Analysis

On each trial the Observer indicated whether the Actor’s eyes were pointing to their left (“Leftward gaze”) or to their right (“Rightward gaze”), relative to a direct fixation. We fit each participant’s proportion of “Rightward gaze” responses (collected when they were in the role of the Observer) as a function of Actor gaze deviation angles across experimental blocks with cumulative Gaussian functions (psychometric function) (Fig. 1b). The 50% point of the psychometric function indicates the gaze deviation angle at which an Observer performs Leftwards/Rightwards gaze classifications at chance level, i.e. the Point of Subjective Direct gaze (PSD) where the Observer perceives the Actor’s gaze as being direct. PSD values that significantly deviate from 0 degrees indicate biased perception of the Actor’s gaze direction. The standard deviation of the underlying Gaussian distribution (SD) provides an estimate of sensitivity to gaze direction, i.e. how capable the Observer is of discriminating different degrees of gaze deviation. Smaller SD values (which correspond to steeper psychometric functions) indicate greater sensitivity to gaze direction. Data analyses were carried out on MATLAB R2016a (https://www.mathworks.com) and JASP 0.8.1.1. (https://jasp-stats.org).

#### Experiment 1

PSD and SD values of 19 participants were submitted to a 2 × 2 Mixed ANOVA, with factors Stimulus Plane (Near Vs Far) and Participant Gender (Female Vs Male). Participant gender was included based on previously observed gender based differences in gaze behaviour directed towards the eye region^14^, which might determine differences in gaze discrimination performance. One participant’s data was discarded due to poor psychometric fits, yielding unreliable estimates of PSD & SD. We also submitted gaze discrimination confidence scores across blocks to an equivalent Factorial ANOVA and correlated confidence scores against SD values. Finally, we analysed head rotation data when participants were in the role of the Actor by correlating within each participant head pitch/roll/yaw rotation data with stimulus deviation angle across trials. Each correlation yielded an r score which described the extent to which head rotation covaried with gaze deviation angle (e.g. whether the head rotated more leftward when viewing a stimulus positioned further in the left visual hemifield). R scores were subsequently submitted to a 2 × 3 Repeated Measures ANOVA with factors Stimulus Plane (Near Vs Far) and Rotation Axis (Pitch Vs Roll VS Yaw), in order to test whether the relationship between head rotation and stimulus deviation varied across Near and Far planes. Since stimuli were prospectively aligned, we would not expect any significant difference in this relationship across Near and Far planes.

#### Experiment 2

We compared SD values of all 18 participants according to two data pooling criteria. A first comparison was based on the plane on which stimuli were displayed on each trial (Objective Plane Comparison). We constructed two psychometric curves related to stimuli presented on either the Near or Far planes, and extracted the resulting SD parameters. SD values were submitted to a 2 × 2 Mixed ANOVA, with factors Stimulus Plane (Objective Near Vs Objective Far) and Participant Gender (Female Vs Male). A second comparison was based on the plane the Observer *thought* stimuli were displayed on in each trial (Subjective Plane Comparison). In this case the psychometric curves were generated based on which plane the Observer believed the stimulus to lie on, on a trial by trial basis. SD values were entered into a 2 × 2 Mixed ANOVA, with factors Stimulus Plane (Subjective Far Vs Subjective Near) and Participant Gender (Female Vs Male).

### Ethics

This study was approved by the UCLIC Research Ethics Committee (UCLIC/1617/003) and was in agreement with the UCL research guidelines and regulations.

## Results

### Experiment 1

A 2 × 2 Mixed ANOVA was run on PSD scores. This showed a non-significant effect of Plane (F(1, 17) = 0.13, p = 0.72., η_p_^2^ = 0.01), a non-significant effect of Gender (F(1, 17) = 0.31, p = 0.59., η_p_^2^ = 0.02) and a non-significant Plane x Gender interaction (F(1, 17) = 0.16, p = 0.69., η_p_^2^ = 0.01). No bias of gaze direction was therefore observed across participants. When analysing SD values, we noticed a violation of the assumption of equality of variance (Levene’s test). We identified two female outliers, with SD scores more than 2 standard deviations above the mean value of the group and proceeded to remove these outliers from the analysis. A Mixed ANOVA on SD values revealed a Main Effect of Plane (F(1, 15) = 4.81, p = 0.04, η_p_^2^ = 0.24), no Main Effect of Gender (F(1, 15) = 1.42, p = 0.25, η_p_^2^ = 0.09) and a non-significant Plane × Gender interaction (F(1, 15) = 1.01, p = 0.33., η_p_^2^ = 0.06). Differences in SD values revealed that participants were more sensitive (smaller SD) to gaze direction information when the Actor fixated on stimuli situated on the same plane occupied by the Observer (Far plane) (Fig. 1c,d). An equivalent 2 × 2 Mixed ANOVA on participant subjective confidence scores only revealed a Main Effect of Plane (F(1, 17) = 4.57, p = 0.047., η_p_^2^ = 0.21): confidence scores were significantly higher in the Far plane, mimicking the pattern of SD values (Fig. 1e). This was further corroborated by a significant SD value/confidence score correlation (r = −0.51, p = 0.001), where greater discrimination sensitivity (smaller SD value) was associated with greater confidence score, thus showing a subjective awareness of higher performance in the Far plane trials. A 2 × 3 Repeated Measures ANOVA on head rotation/stimulus deviation r scores revealed no significant effect of Stimulus Plane or Rotation Axis, and no significant interactions. The lack of a significant Stimulus Plane x Rotation Axis interaction (F(2, 46) = 2.16, p = 0.13., η_p_^2^ = 0.09) indicates that amplitude of head rotations did not vary across stimulus deviations situated on the Near or Far planes. Differences in head rotation cannot therefore account for the difference in gaze discrimination sensitivity reported above.

There are two potential explanations for these smaller SD values observed in the Far plane. The first is that in the Far plane block, Observers’ gaze direction sensitivity was modulated by their knowledge that the virtual stimulus was positioned on either their plane (Far) or the mid plane (Near). A second is that the Observers were able to pick up subtle differences in gaze behaviour directed towards stimuli displayed at different depths. Holograms on the Near and Far planes require different amounts of left and right eye vergence, which in turn could potentially account for differences in gaze discrimination performance (see Fig. S1). A second experiment was specifically aimed at assessing the merit of each hypothesis, where we measured performance as a function of which plane stimuli were factually presented on, or, which plane subjects believed stimuli were displayed on. This enables us to evaluate in isolation participants’ ability of exploiting differences in vergence information to detect the depth plane attended by the Actor, and evaluate the role of assumptions regarding the depth plane the stimulus occupied on gaze discrimination performance.

### Experiment 2

**Figure 2:**
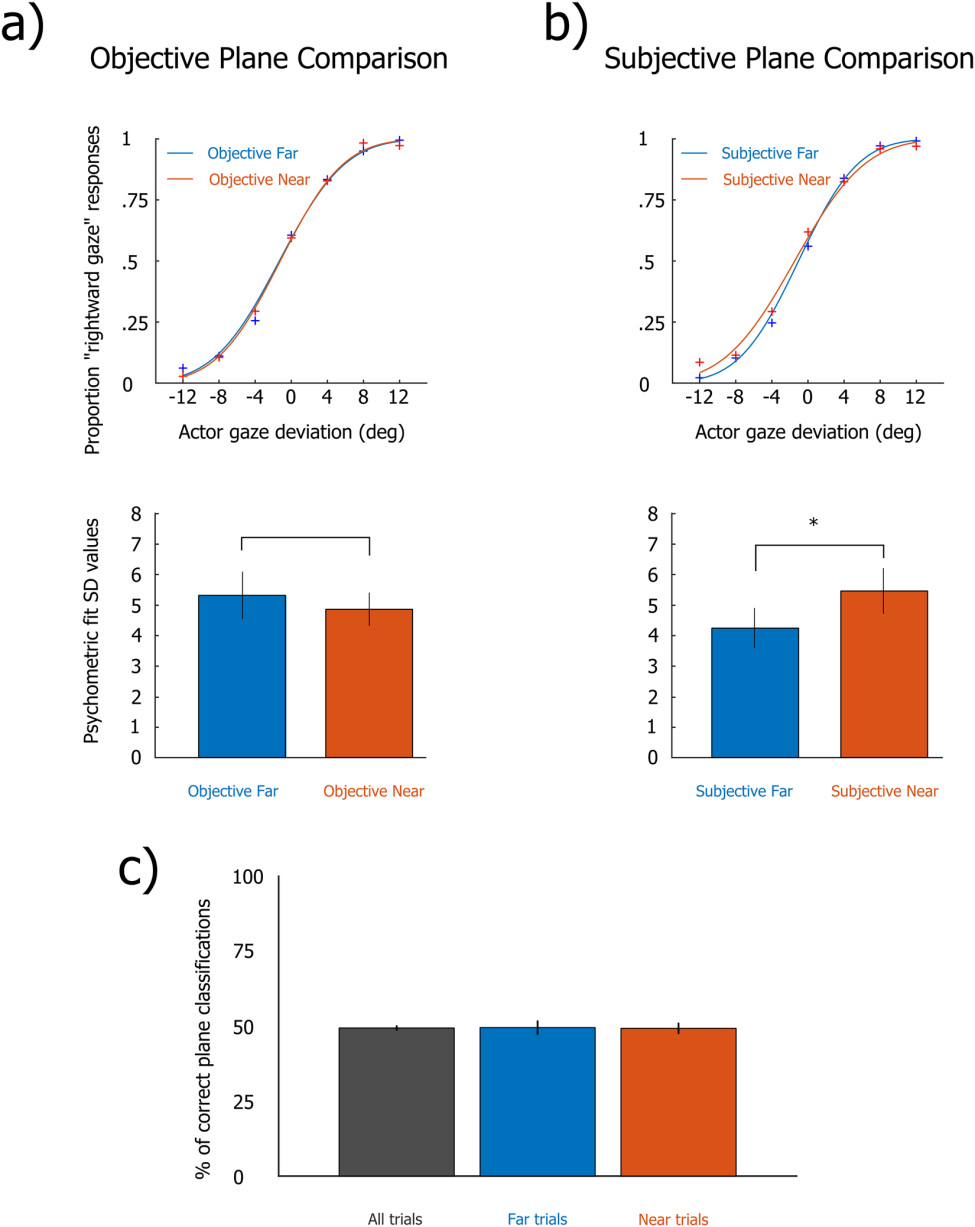
(**a**) Pooled data psychometric fit (top) and average SD values (bottom) for Near and Far plane gaze direction classifications, based on plane on which stimulus was factually presented (Objective Plane Comparison). (**b**) Pooled data psychometric fit (top) and average SD values (bottom) for Near and Far plane gaze direction classifications, based on plane on which participant thought that stimulus was presented (Subjective Plane Comparison). (**c**) Percentage (%) of correct rate of classification across all trials presented on Far & Near planes, or within Far/Near plane trials considered separately.

Given that Experiment 2 was specifically carried out to determine what factor (assumption concerning depth plane where stimulus is displayed on, or, difference in left/right eye vergence across depth planes) explained the differences in gaze discrimination performance (SD) we observed in Experiment 1, here we focused exclusively on testing differences in SD values. We ran two 2 × 2 Mixed ANOVA comparisons on SD values based on the plane on which stimuli were displayed (Objective Plane Comparison; Fig. 2a), or based on the plane the Observer thought the stimulus was displayed on (Subjective Plane Comparison; Fig. 2b) on a trial by trial basis. The Objective Plane Comparison revealed no effect of Plane (F(1, 16) = 0.9, p = 0.36., η_p_^2^ = 0.05) and no significant interaction (F(1, 16) = 0.04, p = 0.85., η_p_^2^ = 0.002). Contrary to Experiment 1, here we observed no significant effect of Gender (F(1, 16) = 2.29, p = 0.15., η_p_^2^ = 0.12). The Subjective Plane Comparison on the other hand, revealed a Main Effect of Plane (F(1, 16) = 4.7, p = 0.046, η_p_^2^ = 0.23) and no significant Plane × Gender interaction (F(1, 16) = 1.22, p = 0.28, η_p_^2^ = 0.07). This suggests that a subjective expectation regarding the depth plane positioning of the stimulus attended by the Actor modulated gaze discrimination performance in the Observer. We also tested rate of correct plane classifications: a binomial test revealed that participants operated at chance level performance (49% of correct classifications; p = 0.55, two-sided) when evaluating whether the Actor was fixating on a stimulus presented on the Near or Far plane. We also ran binomial tests between Objective and Subjective across depth planes taken separately, which confirmed that participants operated classifications at chance level performance (Near binomial p = 0.63; Far binomial p = 0.73). Taken together these results clearly show that participants were incapable of reliably detecting, and therefore exploiting, differences in the Actor’s left/right eye vergence across depth conditions to inform gaze direction classifications.

Binomial tests of participants’ ratio of Near/Far plane responses in Experiment 2 also showed that the majority of participants (14 out of 18) equally distributed number of plane classifications, thus showing no prior bias towards one or the other plane. The 4 remaining participants showed biased classifications, but not in a consistent direction: 2 were biased in favour of the Near plane, 2 in favour of the Far plane. These data show no strong overall prior expectation concerning an AR user’s focus of attention under conditions of visual uncertainty. This implies that expectations of AR user’s focus of attention can be influenced by contextual cues or prior experience.

## Discussion

We investigated gaze judgments in the context of dyadic interactions mediated by AR head mounted interfaces, and assessed sensitivity to gaze directionality as a function of expectations concerning the depth plane positioning of virtual content attended by an AR user. Experiment 1 revealed steeper psychometric functions (smaller SD), indicating greater gaze direction sensitivity, and improved discrimination performance, when participants were explicitly informed that an AR user was fixating on virtual stimuli situated on the plane occupied by the participant (Far plane), as opposed to being positioned halfway between the AR user and the participant (Near Plane). This improvement in performance was mirrored by participants’ gaze classification subjective confidence scores, where higher subjective ratings were reported in the Far plane blocks, thus linking self-efficacy to low level visual discrimination performance^17^. We observed no systematic biases in gaze direction estimates as a function of depth plane (non significant difference in PSD). Experiment 2 showed that the modulatory effect on gaze discrimination performance (SD values) was explained by participants’ expectations concerning the plane occupied by the stimulus the AR user was focusing on. Enhanced gaze direction sensitivity was observed in instances in which participants *believed* (Subjective Plane Comparison) that the actor was focusing on stimuli situated on their plane (Far) as opposed to the mid plane (Near), replicating the finding of Experiment 1. Importantly, this was independent of their beliefs being correct, as evidenced by chance level rate of plane classifications, and a non-significant difference in gaze discrimination performance between stimuli factually presented on the Near or Far planes (Objective Plane Comparison): participants were incapable of exploiting, differences in vergence information across depth planes to inform gaze direction classifications, and therefore performance shifts were driven by assumptions as to which plane stimuli were displayed on. These results demonstrate that gaze interactions mediated by AR technologies are modulated by expectations concerning the positioning of virtual content attended by an AR user. An external viewer’s ability of determining an AR user’s gaze is improved (or impaired) by the awareness that the AR user is focusing on stimuli projected on their (or a different) depth plane.

Direct gaze provides a strong biological signal, which expresses interest or hostility and cues social behaviour^1,18^. Direct gaze is also known to enhance cognition and attention, i.e. the ‘eye contact effect’^19,20^. While in most circumstances direct gaze fairly unambiguously signals interest in the recipient, use of AR HMD interfaces introduce elements of uncertainty that impair a clear evaluation of the AR user’s focus of attention. The use of AR in social contexts necessarily entails asymmetries in visual awareness of holographic content between an AR user and an external observer. When an AR user looks in our direction, we can either interpret that behaviour as directed towards us, or towards augmentations positioned on our line of sight, which we are visually unaware of. This visual asymmetry, and the ambiguity it entails, are unique to real world interactions mediated by AR HMDs.

Experiments 1 and 2 showed that expectations concerning the positioning of virtual stimuli attended by an AR user can modulate gaze discrimination performance. In Experiment 1 we manipulated these expectations by providing the Observer with an explicit awareness of which plane the Actor fixated on. In Experiment 2, we adopted a data-driven approach where trials were pooled according to subjective estimates of which plane the Actor was thought to be fixating on. Both Experiments showed improved gaze determination based on an awareness (Experiment 1), or assumption (Experiment 2), that the Actor was fixating on a virtual stimulus positioned on the participant’s plane.

A possible explanation for this enhanced performance can be found in the social cognition literature, where information about a gazer’s mental state can affect gaze processing^21^. Using a deception technique, Teufel and co-workers (2009) manipulated participant’s beliefs that a confederate’s view was obstructed by opaque glasses and showed that attributions of mental state exert a top-down modulatory effect on gaze direction acuity. This provided clear evidence that sensory coding of a gaze cue’s physical characteristics can be top-down modulated by mental-state attribution^21^. Studies investigating gaze dependent autonomic responses^22^, evoked brain activity^23^ and reflexive attentional responses^24,25^, similarly suggested that attributions of mental state overlap with processing of gaze information. Extrapolating from this literature we could for example hypothesize that gaze processing might interact with Observer’s assumptions of how clearly the Actor can see him/her. When participants had the opportunity of viewing stimuli in the Hololens prior to running the experiment, an aspect that could be noticed was that when viewing holograms on the Near plane, the other participant appeared out of focus (due to eyes verging and accommodating to stimuli at a different depth than the other participant), whereas when viewing stimuli on the Far plane, the other participant was clearly visible. As in one-way mirror deception studies^21^, where gaze discrimination improved when participants believed the gazer could see them, here we could say that gaze discrimination improves when participants believe the gazer is able of seeing them more clearly. An alternative explanation is that improved performance is caused by observer’s expectations of the Actor’s focus of attention. The Observer’s expectation that the Actor’s attention is focused on a point closer to him/her, and is perhaps more aware of him/her, might improve performance. Both of these possibilities would be instances of mental state attribution as they relate to participant’s beliefs of what the other is experiencing.

Another possibility accounting for these findings is that gaze discrimination is improved when eyes converge on a stimulus closer to us, irrespective of mental state attribution. We could hypothesize for instance that stimuli within peripersonal space recruit more attention, and that gaze signals directed towards these stimuli are processed with higher precision. There are crossmodal integration studies for example that show enhanced attention to looming stimuli, approaching peripersonal space^26,27^. In a conceptually similar way, we could say that the presumed positioning of a virtual object closer to the Observer enhances attention, and this in turn reflects on gaze discrimination accuracy. Previous work has however shown comparable discrimination performance for avatar stimulus gaze deviations centred around the observer (centred on the participant plane), or averted with respect to the observer (falling beyond the participant plane)^8^. While this would suggest no modulatory effect of fixations converging on Vs falling beyond peripersonal space on gaze sensitivity, this does not necessarily discount the possibility that a mechanism of this type might occur in real world dyadic interactions involving virtual stimuli. Studies involving one-way deception techniques, as in Teufel *et al*.^21^, or studies involving non-anthropomorphic neutral stimuli would be required to unequivocally determine the underlying mechanism driving these effects.

These findings highlight more in general how conditions of sensory uncertainty can accompany the use of specific forms of technology, with measurable impacts on gaze determination. We have highlighted this in the context of gaze interactions mediated by AR devices. The development of devices such as the Microsoft HoloLens, the DAQRI Smart Glasses and the Magic Leap, evidence that AR technologies are increasingly becoming important tools in everyday tasks and work activities. With AR a user is immersed in a 3d environment where virtual and real content are properly registered, thus offering an opportunity to leverage the natural association between spatial cognition, attention, memory and response selection^28^. AR allows interaction (sampling, inspection and manipulation) of virtual content based on the cognitive and motor repertoire we adopt in our everyday environment: i.e. we can inspect an object by walking around it and appreciate it in finer detail by getting closer. These features highlight the advantages of AR over more traditional forms of assistive technology. However, our results also show that the use of such technologies carry an inherent element of visual uncertainty, that can negatively impact people’s ability of accurately evaluating the AR user’s gaze behaviours. One can appreciate the costs of reduced gaze determination in collaborative work environments, when considering the role of gaze in guiding cooperative behaviours and signalling the presence of potentially harmful environmental stimuli. For example, if we assume that an AR user’s gaze behaviours are directed at augmentations which happen to fall on our line of site, these behaviours might be less effective at cueing our attention towards joint-task relevant information or warning us of the spatial location of environmental hazards. These results therefore further our understanding of the impact of technology on social behaviour and gaze processing and can provide insights for the design of AR interfaces that reduce the sources of visual uncertainty that normally accompany the use of these technologies.

## Conclusion

We studied gaze interactions mediated by an Augmented Reality (AR) headset in participant pairs, and evaluated the role of expectations concerning the positioning of virtual content attended by an AR user on gaze perception in two psychophysical experiments. The first experiment showed that gaze discrimination performance was improved when a participant was aware that the AR user was focusing on stimuli positioned on the participant’s plane, as opposed to being positioned halfway between the AR user and the participant. The second experiment showed that this modulatory effect was explained by participants’ expectations concerning which plane the AR user was focusing on, irrespective of this assumption being correct. If we assume that an AR user’s attention is not directed at us, but towards augmentations positioned somewhere in between us, we might be less capable of extracting behaviourally relevant information (e.g. location of joint task items or presence of environmental hazards) signalled by their gaze behaviours.

## Supplementary information

Fig S1

Supplementary Dataset 1&2
