# Supplementary material for: Assumptions about the positioning of virtual stimuli affect gaze direction estimates during Augmented Reality based interactions
Source: Sci Rep. 2019 Feb 22;9:2566. doi: 10.1038/s41598-019-39311-1 (PMC6384932; doi:10.1038/s41598-019-39311-1)
Supplement: Supplementary file 1 — Fig S1 [file 41598_2019_39311_MOESM1_ESM.pdf]

Assumptions about the positioning of virtual stimuli affect gaze direction estimates during Augmented Reality based interactions

Nicola Binetti[1], Tianchang Cheng[1], Isabelle Mareschal[2], Duncan Brumby[1], Simon Julier[3] and Nadia Bianchi-Berthouze[1]

[1]UCL Interaction Centre, University College London, UK

[2]School of Biological and Chemical Sciences, Psychology, Queen Mary University of London, UK

[3]Department of Computer Science, University College London, UK

Corresponding author: nicolabinetti@gmail.com

Supplementary information

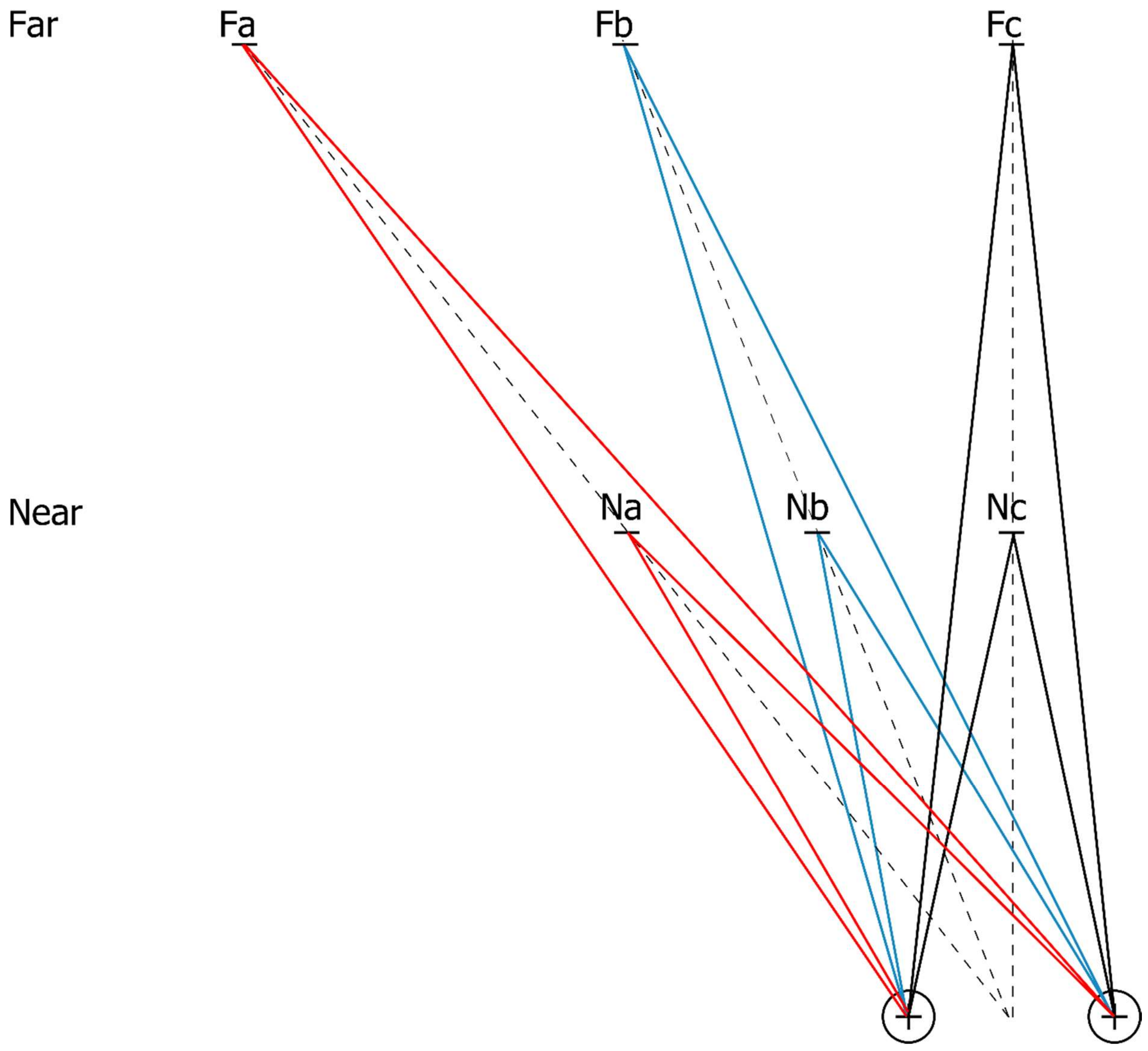

Fig S1: Considering that left / right eyes are offset from the midline, vergence angles will slightly differ when fixating on an equivalent stimulus positioned on the Near or Far planes. From a geometrical standpoint the vergence angles should be more pronounced for stimuli on the Near plane: eg fixating at stimulus ‘a’ on the left requires a greater inward rotation of the right eye when it is on the Near plane (Na) as opposed to when it is on the Far plane (Fa). If participants were able of detecting these differences in vergence angles between N and F planes, we would expect improved performance (steeper psychometric functions) for Actor fixations produced on the Near plane.
